# Unexpectedly Rapid Onset of Severe Sarcopenia in an Elderly Diabetic Man following SGLT2i Administration: A Case Report

**DOI:** 10.3390/jcm13102828

**Published:** 2024-05-11

**Authors:** Paulina Czarnecka, Kinga Czarnecka, Olga Tronina

**Affiliations:** Department of Transplantology, Immunology, Nephrology and Internal Diseases, Medical University of Warsaw, 02-006 Warsaw, Poland

**Keywords:** diabetes mellites, SGLT2i, sarcopenia

## Abstract

Sarcopenia is characterized by the progressive loss of muscle mass, strength, and function and poses a significant health challenge among people with diabetes. Sodium–glucose cotransporter-2 inhibitors (SGLT2is) are the backbone of type 2 diabetes treatment. The interplay between SGLT2is and sarcopenia is an area of active research with inconclusive results. This article presents an unexpectedly rapid weight reduction, along with physical performance deterioration, in an elderly patient with type 2 diabetes, which led to treatment discontinuation. A bioelectrical impedance analysis confirmed severe sarcopenia development. Until more data are available, sarcopenia and body composition screening and monitoring may be warranted whenever SGLT2is are prescribed.

## 1. Introduction

Sarcopenia is a progressive skeletal muscle condition defined by low muscle mass, strength, and physical performance, with the latter being indicative of the sarcopenia severity [1]. Sarcopenia is more prevalent in people suffering from type 2 diabetes mellitus (T2DM) compared with the general population [2].

Notably, sarcopenia is categorized as one of the three most disabling complications in T2DM, leading to frailty, a diminished quality of life (QoL), falls, fractures, and increased mortality [3,4,5,6,7]. There is a bidirectional interplay between T2DM and sarcopenia [7]. The latter may have a deleterious influence on glycemic control, wherein T2DM entails a chronic inflammation state; oxidative stress; and protein breakdown in muscles, which potentiates muscle mass loss, which may trigger the onset of sarcopenia [8].

Sodium–glucose cotransporter-2 inhibitors (SGLT2is), also known as flozines, are one of the first-line agents for type 2 diabetes mellitus treatment [9]. Their main mechanism of action is to promote renal glucose excretion through SGLT2 cotransporter blockage in the proximal tube, which further manifests as glucosuria.

The use of flozines may lead to body weight (BW) reduction, which is typically welcome in diabetic patients, especially when they are obese or overweight. Nevertheless, there is evidence to suggest that BW reduction may also entail muscle mass (MM) reduction, potentially putting the patient at risk of sarcopenia [10,11,12].

This paper reports the unexpectedly rapid development of severe sarcopenia in a patient secondary to SGLT2i intake, which led to treatment discontinuation. Understanding the relationship between SGLT2is and sarcopenia is crucial for optimizing diabetes treatments while also safeguarding muscle health.

## 2. Case Description

A 72-year-old man with a decade-long history of diabetes mellites type 2 was referred to a nephrologist because of randomly identified proteinuria in spot urinalysis. His medical history included acid reflux, benign prostatic hyperplasia, and hypertriglyceridemia, all adequately controlled with medications (a complete list of medications is provided in Table 1).

At the time of T2DM diagnosis, the patient was started on metformin (a total daily dose of 3 g), which was poorly tolerated because of gastrointestinal symptoms; hence, he was transitioned to gliclazide. In the past 2 years, his diabetes treatment was stable and consisted of gliclazide (60 mg) and linagliptin (5 mg). At the time of his first visit, his blood pressure was 120/70 mmHg, with a regular heart rate of 60 beats per minute. There was no evidence of edema or fluid retention. His weight was 80 kg, and this height was 174 cm.

A laboratory investigation revealed stage 2 chronic kidney disease (CKD) with an estimated glomerular filtration rate (eGFR) of 67 mL/min/1.73 m^2^; spot urine proteinuria of 100 mg; fasting glucose of 140 mg/dL; HbA1c of 8.5%; total cholesterol of 156 mg/dL; LDL of 70 mg/dL; and triglycerides of 134 mg/dL. The remaining results were unremarkable. A detailed report is provided in Table 1. The subject regularly exercised 3–4 times a week for a total of 180 min minimum. Nutritional guidance was also provided and included regular meal consumption, salt intake not exceeding 5 g daily, carbohydrates of a low glycemic index to be favored (approximately 45% of daily calorie intake), protein intake not to exceed 1 g/kg body weight, saturated rich fat < 10% of daily calorie intake, and fiber intake being approximately 25 g daily.

A staggered approach was employed with regard to proteinuria evaluation with this patient. Considering his suboptimal diabetes management, the first steps were directed towards glucose control improvement. There was no evidence of excessive subcutaneous fat tissue, and his musculature was quite well developed.

Despite a BMI that was indicative of overweight (26.4 kg/m^2^), we were hesitant to recommend major weight reduction at this point and decided to evaluate the patient more thoroughly before making such a decision. Bioelectrical impedance analysis (BIA) was scheduled for the following week. The patient’s diabetes management was re-evaluated by substituting gliclazide and linagliptin with dapagliflozin (10 mg). Potential adverse reactions were discussed, and the treatment was approved by the patient.

The subject presented for a follow-up visit a month later. He was hemodynamically stable with a 78 kg (−2 kg) body weight. Kidney function and electrolytes were stable (for details, refer to Table 1). BIA was performed 10 days following dapagliflozin initiation and revealed a fat mass (FM) of 20.9 kg (26.1%), an MM of 25.5 kg (31.9%), and an appendicular skeletal muscle mass index (ASMI) of 8.39 kg/m^2^. The patient was tolerating the treatment well; his glucose levels were improving, with fasting glucose at 90 mg/dL, and random glycemia did not exceed 140 mg/dL in at-home self-assessments. There was no evidence of urinary tract infection, and spot urine protein had decreased to 50 mg. His C-peptide was 3.68 pg/mL. Treatment with dapagliflozin was maintained until the next visit approximately 2 months later.

The patient came in for the appointment with complaints of fatigue, which was interfering with his regular physical activity schedule and had started to interfere with activities of daily living (ADLs). He consulted his primary care physician prior to the visit due to the above-mentioned symptoms. There was no additional work-up recommended. His weight had reduced further to 74 kg. He was hemodynamically stable, with no evidence of infection of any kind. His blood glucose levels continued to improve, with a fasting glycemia of 86 mg/dL on average, a random value not exceeding 130 mg/dL, and an HbA1C of 6.8%. No hypoglycemic episodes were observed. His kidney function remained stable, as did electrolytes, and the spot urinalysis was negative for proteinuria. Laboratory investigations revealed latent hypothyroidism (thyrotropin 9.5 IU/L and free hormones within normal range), and hormone supplementation was initiated (levothyroxine 12.5 ug). Additional investigations ruled out euglycemic ketosis (both urine and blood ketones were within normal ranges). Calf circumference was used as a screening tool for sarcopenia, but this value was above the cutoff value for a sarcopenia diagnosis (33.5 cm).

In light of these findings, imaging and endoscopy work-ups were recommended to exclude neoplasm as an underlying cause of the rapid weight loss and fatigue. Diabetic therapy was maintained with an expedited follow-up visit in a month or sooner if the recommended work-up was completed early.

The subject presented at the office 5 weeks later: his weight loss had continued, and his BW was 70.2 kg. His ADLs were severely impacted. However, the prescribed work-up was negative for neoplasm.

The calf circumference was 33 cm and, therefore, above the threshold for a sarcopenia diagnosis, but given that the fatigue was markedly impacting the patient’s QoL, it was decided to repeat BIA (FM, 21.8 kg—31.1%; MM, 21.0 kg—30%; and ASMI, 6.92 kg/m^2^) and perform a physical performance check. Gait speed was measured, with a result of 0.74 m/s. Based on these reports, severe sarcopenia was diagnosed.

Having discussed the results with the patient, it was decided not to continue the SGLT2i treatment despite all assumed potential benefits. The patient was transitioned to gliclazide, at a higher dose than previously prescribed (120 mg), and linagliptin, with a recommendation to closely monitor glycemia. The subject was provided with nutritional and resistance training guidance.

In a month, his weight improved to 72 kg, and his fatigue also improved. Glycemia control was satisfactory. There was no evidence of proteinuria relapse, and the kidney function was preserved.

Six months following dapagliflozin cessation, the subject restored his body weight to 74 kg but claimed that his physical performance was inferior compared with baseline. At a one-year follow-up, the subject was found to have recovered (with sequel) from severe sarcopenia. BIA was repeated and revealed an MM of 23.6 kg (31% of BW), an FM of 21.7 kg (28.5% of BW), and an ASMI of 7.76 kg/m^2^. However, his physical performance continues to be inferior compared with the baseline.

## 3. Patient Perspective

In the patient’s view, he was referred to the nephrologist because of laboratory abnormalities that were not accompanied by any symptoms. Therefore, despite recognizing the potential long-term benefits of the recommended treatment and the notable improvement in laboratory reports over time, he felt that his QoL and ADLs deteriorated significantly, which was disproportionate to the benefits.

The patient confirmed that the need for additional and frequent appointments at the nephrology office, triggered by the onset of SGLT2i-attributed complications, was time-consuming and quite burdensome from an organizational standpoint, as he resides far from the clinic and needed family members to be involved. Simultaneously, he underscored that he was motivated to attend all the appointed visits and work-ups, as he felt that he was treated with the utmost care and diligence, which was lacking at the primary care level. He recounted that the symptoms of fatigue and weight loss that he initially reported to a general practitioner were neglected and attributed to aging, with no follow-up investigation to determine the reason for the rapid symptom onset and progression.

## 4. Discussion

The present report depicts a severe case of sarcopenia secondary to SGLT2i administration. This case documents an unanticipatedly rapid decrease in the subject’s BW and MM, leading to his performance significantly worsening.

The presented paper reports a BW loss of virtually 10 kg over 4 months. Several meta-analyses have previously demonstrated that SGLT2is may exert BW reduction properties, and BW loss may entail an MM decrease, which supports our observation [13,14]. Importantly, the literature remains inconclusive as to whether SGLT2is impact MM, as other authors observed MM increases following SGLT2 intake or found a negligible effect [15,16,17,18,19,20].

The documented reduction in MM accounted for approximately 40% of the patient’s total BW loss. Zhang et al. previously found that the lean mass (LM) may be responsible for approximately one-third (10% to 40%) of weight loss following SGLT2i intake [14,21]. Bolinder J reported similar results [22]. These observations are broadly consistent with our findings regarding the extent of MM loss. Of note, behavioral measures, including diet and regular physical exercise, that lead to BW reduction engage a similar proportion of body compartments, which can lead to a one-third MM reduction [23,24]. Contrary to our observations, others have reported that BW reductions should not exceed 2–3 kg, but this may be highly dependent on the type of SGLT2i used and its dose [10,11,12,25]. Yabe et al. investigated empagliflozin’s impact on weight loss and composition in an elderly T2DM Japanese population. Within the 52-week observation period, muscle mass and gait remained preserved [26]. To some extent, the discrepancies between the results presented by Yabe et al. and the current study may be explained by an increased calorie intake in the empagliflozin arm, which could have added to the maintenance of muscle mass. At this point, it is unclear whether all flozines have similar safety profiles in terms of sarcopenia development. Available data documenting BW reduction and the extent of MM reduction are scarce and mainly come from secondary outcome analyses [11,27]. There is significant heterogeneity in terms of the modalities used to investigate body composition and populations studied, which may result in inconclusive results. Furthermore, most studies are not adjusted for concomitant medication. Therefore, more data are needed to elucidate to what extent flozines trigger MM reduction.

The initiation of dapagliflozin resulted in significant glycemic control improvement. Additionally, within the first month of dapagliflozin intake, a desirable weight loss of 2 kg was observed in the patient. However, the pace of continued weight loss was alarming. It has been recognized that the impact of flozines on body composition may change over time. Initially, the BW reduction can mostly be attributed to fluid and calorie loss, given their primary mechanisms of action. However, with time, regulatory mechanisms step in, and BW reduction should plateau after 26 weeks of treatment [28]. Noteworthy, rapid, and intense weight reductions over a short period may have deleterious metabolic consequences, including increased liver steatosis. In the present case, the SGLT2i intake was limited to 4 months, so it is difficult to speculate regarding mid- and long-term SGLT2i outcomes. The dynamics of BW alteration did not warrant further flozine use in this particular patient.

It is also important to note that flozines are recommended as a first-line treatment in populations including patients with CKD and HF and can be prescribed over a patient’s lifetime [29]. Most of the studies that have investigated the body-composition-modifying properties of flozines have been limited to 24-week follow-ups. More studies are needed to understand their long-term outcomes in the T2DM population.

In the presented case report, sarcopenia was diagnosed based on a BIA, and a computed tomography (CT) scan was not assessed for body sarcopenia. The imaging was carried out outside of our institution, and only the CT scan report was provided by the patient. The limitations of using BIAs to determine body composition are well established and include an assumption of fixed hydration [30,31]. This may have an impact if comorbidities like CKD or heart failure (HF) are present. More precise methods, such as magnetic resonance imaging (MRI) or CT, continue to be a gold standard for sarcopenia diagnosis. However, high cost and low accessibility prevent them from being applied in routine clinical practice [32]. Nevertheless, the authors believe that using consistent body composition assessment methods allowed us to draw meaningful conclusions on the diagnosis of sarcopenia in the presented case.

The subject was not specifically evaluated for sarcopenia before SGLT2i initiation; however, the BIA at baseline confirmed that the subject was not sarcopenic at that point. Regular body composition assessments allowed for a relatively early diagnosis of sarcopenia onset and prevented potentially serious consequences of continued SGLT2i usage in this patient. Until more evidence is presented and the risk factors for sarcopenia onset following flozine usage are identified, the authors are of the opinion that patients who are considered for SGLT2is may benefit from sarcopenia status verification before prescribing SGLT2is, with periodic verification thereafter. In patients at high risk of sarcopenia, a regular body composition assessment may be warranted. At our clinic, quarterly sarcopenia surveillance has already been initiated in patients who are started on SGLT2is in addition to baseline sarcopenia screening.

Notably, older patients may not be the only ones at risk. Flozine usage has also been extended to non-diabetic patients with CKD and HF, in whom BW reduction may not always be desirable. Heart failure and CKD populations have a relatively high sarcopenia burden, which may be further amplified by SGLT2i usage, notwithstanding its beneficial cardiovascular and kidney outcomes. When a relatively young patient with underlying chronic bowel disease receives flozines for CKD, this may trigger unintentional BW loss and disability without closely monitoring their nutrition and health status. There are no recommendations to include sarcopenia screening prior to prescribing SGLT2is.

In the present case, we discussed all the potential risks and benefits with the patient and his family, which they acknowledged and accepted. Diabetes mellitus management in the elderly population is much more complex than glycemic control and micro- and macrovascular complication management; it constitutes a complex task, with a fair number of contributing factors that need to be considered. This patient report provides us with a significant insight into the discrepancies between therapy goals from the perspectives of both the treating physician and the patient. While recognizing the benefits of the prescribed treatment, patient-specific factors and preferences should always be taken into account in the decision-making process.

While recognizing the multiplicity of potential benefits of SGLT2is, including weight loss, it should be borne in mind that flozines have not been on the market long enough to elucidate all their potential adverse reactions. In this case, the authors would like to emphasize the necessity of a tailored approach to type 2 diabetes management and closer therapy surveillance. The presented case suggests that it may be beneficial to implement sarcopenia screening when SGLT2is are considered. Additionally, regular body composition surveillance may be warranted in case of increased sarcopenia risk at baseline until we better understand the dynamics of BW following flozine intake and can discriminate the patient profiles that would benefit from them the most.

## 5. Conclusions

This case highlights the need for an individualized approach to managing diabetes while also safeguarding muscle health and having a heightened awareness of sarcopenia in elderly patients receiving SGLT2 inhibitors. Collaborative multidisciplinary efforts among nephrologists, endocrinologists, and general practitioners are essential. Further research is warranted to elucidate the relationships and mechanisms underlying SGLT2i-associated sarcopenia and the development of targeted interventions.

## Figures and Tables

**Table 1:** Laboratory results and concomitant medication over time.

Lab result (normal ranges)	Baseline	4 weeks	12 weeks	17 weeks
AST, U/L; (1–40)	19	-	21	-
ALT, U/L; (10–55)	25	-	18	-
sCr, mg/dL; (0.7–1.2)	0.9	1.1	1	0.9
Albumin, mg/dL; (3.5–5.3)	4.2	-	4.4	-
Glucose fasting, mg/dL	140	90	86	-
HbA1c, %	8.5	-	6.7	6.5
TSH, IU/L; (0.27–4.2)	9.5	5.74	3.72	-
fT4, ng/dL; (0.93–1.7)	0.96	-	-	-
C-peptide, ng/mL; (0.78–5.19)	1.83	-	-	-
Total cholesterol, mg/dL	156	-	-	-
LDL, mg/dL	70	-	60	-
TG, mg/dL	134	-	141	-
K, mmol/L; (3.6–5.1)	4.3	-	4.4	3.9
Na, mmol/L; (135–145)	136	-	136	139
Ca, mg/dL; (8.8–10.2)	9.1	-	9.0	-
Vitamin D, ng/mL; (30–80)	47.15	-	-	-
Ketones urine	negative	-	negative	-
Ketones blood; (<0.6 mmol/L)	-	-	<0.6 mmol/L	-
Proteinuria, mg	100	50	negative	negative
Concomitant medication/daily dose	Baseline	4 weeks	12 weeks	17 weeks
Fenofibrate, mg	160	160	160	160
Gliclazide, mg	60	-	-	120
Linagliptin, mg	5	-	-	5
Finasteride, mg	5	5	5	5
Vitamin D, U	2000	2000	2000	2000
Acetylsalicylic acid, mg	75	75	75	75
Pantoprazole, mg	20	20	20	20
Levothyroxine, ug	Started on 12.5	12.5	12.5	12.5
Dapagliflozin, mg	Started on 10	10	10	discontinued

AST, aspartate aminotransferase; ALT, alanine aminotransferase; sCr, serum creatinine; HbA1c, glycated hemoglobin; TG, triglycerides; TSH, thyrotropin.

## Data Availability

Data are contained within the article.
